# Outcome after modified Putti-Platt procedure for recurrent traumatic anterior shoulder dislocations

**DOI:** 10.1007/s11751-013-0171-x

**Published:** 2013-08-09

**Authors:** Gijs I. T. Iordens, Esther M. M. Van Lieshout, Bernd C. Van Es, Niels W. L. Schep, Roelf S. Breederveld, Peter Patka, Dennis Den Hartog

**Affiliations:** 1 Department of Surgery-Traumatology, Erasmus MC, University Medical Center Rotterdam, PO Box 2040, 3000 CA Rotterdam, The Netherlands; 2 Trauma Unit Department of Surgery, AMC, Amsterdam, The Netherlands; 3 Department of Surgery, Red Cross Hospital, Beverwijk, The Netherlands

**Keywords:** Shoulder, Instability, Bankert, Putti-Platt, Procedure

## Abstract

Most recent studies on procedures for stabilizing the glenohumeral joint focus on arthroscopic techniques. A relatively simple open procedure is the modified Putti-Platt procedure. The aim of these retrospective case series was to evaluate the functional outcome, patient satisfaction, and quality of life of patients who underwent this procedure. After a median follow-up time of 4.7 (P_25_–P_75_ 1.7–6.8) years, fifty-one patients could be enrolled with a mean age of 25 (21–39) years. Five patients (10 %) reported re-dislocations. The median Constant score for the affected side was 84 (P_25_–P_75_ 75–91). Median loss of motion in abduction, elevation, external rotation, and external rotation in 90° of abduction did not exceed 10° when compared to the healthy shoulder. A median Rowe score of 92 (P_25_–P_75_ 75–95) was measured. The WOSI score and SF-36 showed excellent quality of life. The VAS proved high patient satisfaction with the outcome; 7.9 (6.8–9.5). We concluded that the modified Putti-Platt procedure leads to excellent outcome scores and only marginal restriction in range of motion combined with a high patient satisfaction. Our data prove that excellent results can be obtained with a relatively simple open procedure.

## Introduction

The incidence of recurrent instability after a first-time shoulder dislocation ranges from 10 % in patients older than 40 years to almost 90 % in patients younger than 20 years [1–3]. To durably treat an unstable shoulder, it requires surgical intervention. Despite the growing experience with arthroscopic techniques, open procedures still render similar results [4–6]. One of the oldest open “non-anatomic” techniques for this purpose is the Putti-Platt procedure. This procedure was designed to shorten the subscapularis muscle and the anterior capsule in order to stabilize the glenohumeral joint. However, underlying pathologic lesions like a labral tear is not addressed [7–9]. This method frequently resulted in significant loss of external rotation and concomitant osteoarthritis [8, 10, 11]. During the past two decades, several modifications of the original Putti-Platt procedure have been developed [12–15]. Since 2000, a specific modification of the Putti-Platt procedure is the preferred treatment for recurrent anterior glenohumeral instability in one academic and one nonacademic teaching hospital in The Netherlands. This modification implies imbrication of the subscapularis muscle and capsule along with anatomic repair of underlying pathology.

Most studies on modified Putti-Platt procedures focused on recurrent instability and functional outcome. Data on patient satisfaction with the outcome and quality of life after a modified Putti-Platt procedure are not available. The aim of this retrospective study was to evaluate these specific aspects of recovery as well as the functional outcome after a modified Putti-Platt procedure for recurrent shoulder instability.

## Materials and methods

### Patients

This is a retrospective case series including all adult patients (aged 18 years or older) that were treated with a modified Putti-Platt procedure after recurrent (i.e., two or more) anterior shoulder dislocation between 2000 and 2010. All consecutive patients were selected from two hospital databases, one academic center, and one teaching hospital. This procedure was standard care in the treatment of recurrent anterior glenohumeral instability in both clinics. Patients with insufficient comprehension of the Dutch language to complete the questionnaires were excluded. All patients gave written informed consent to participate in this study, which was approved by the medical research ethics committees of both participating hospitals.

### Surgical procedure

An approximately 7 cm incision from slightly lateral to the coracoid process was made running along the deltopectoral groove. The posterolateral surface of the humeral head was carefully inspected by palpation for the presence of an impression fracture (Hill-Sachs lesion). After exposing the tendon of the subscapularis muscle, it was vertically transected, 1–2 cm proximal from its insertion on the minor tubercle. After incising the capsule, a Fukuda retractor was used facilitating inspection of the glenoid surface. When a labral detachment (i.e., Bankart lesion) was identified, it was repaired in the following way. The labrum was reattached to the anterior glenoidal rim together with the capsule using suture anchors. In case of a capsular tear, a capsular repair was performed. Subsequently, the subscapularis muscle was shortened by transferring the medial part under the lateral part; consequently, imbrication of the capsule was achieved in all patients. Over-tightening of the subscapularis muscle may lead to an undue post-operative restriction in range of motion. In order to asses whether the subscapularis muscle and capsule were not too tight or too loose, the arm was placed alongside the body with the elbow in 90° flexion with the thumb pointing up during subscapularis reefing. In this position, the shoulder was required to reach neutral position (0° of external rotation). However, when unsupported, gravity was not expected to externally rotate the arm any further. It should be noted that the lateral stump of the subscapularis muscle was not attached to the anterior glenoid rim as customary in the original Putti-Platt procedure. This modified Putti-Platt procedure, which was used for all patients in this study, has also been described elsewhere [12–14]. Post-operatively, all patients received an immobilizing sling (e.g., Polysling or Gilchrist). Between weeks two and six, only circumduction exercises were allowed. From 6 weeks onward, patients were permitted external rotation and strength-enhancing exercises if tolerated.

### Outcomes assessment and data collection

The shoulder function was assessed primarily using the Constant score. This scoring system consists of four variables, reflecting function, range of motion, pain, and strength of the shoulder joint [16]. A secondary functional outcome measures were the disability of arm, shoulder, and hand (DASH) score. Scores ranged from zero points (representing no disability) to 100 points (representing severe disability) [17–19]. In addition, the Rowe score was used. This is a tool for the assessment of shoulder instability after shoulder-stabilizing procedures [20]. The range of motion (ROM) at the time of follow-up was measured using a goniometer. Furthermore, the injury-related quality of life was assessed using the Western Ontario Shoulder Index (WOSI). It was calculated as a percentage of the maximum possible score, with a higher score indicating less quality of life [21]. The health-related quality of life was measured using the Short Form-36; the scores for the physical and mental components were converted to a norm-based score and compared with the norms for the general population of the United States [22]. Patient satisfaction with the outcome of treatment was measured using a visual analog scale (VAS), in which zero indicated full dissatisfaction and 10 indicated full satisfaction.

Data were collected from medical charts and a questionnaire completed by the patients. Baseline data included age at the time of surgery, dominant side, gender, tobacco and alcohol consumption, and medical history at the time of surgery. Injury-related variables included initial trauma mechanism, affected side, duration of instability, number of dislocations before surgery, previous stabilizing procedures, and presence of a labral lesion or Hill-Sachs lesion. Intervention-related variables included number of anchors used, institution, post-operative treatment, and duration and type of immobilization and physical therapy. All intra- and post-operative complications and secondary interventions were recorded.

### Statistical analysis

Data were analyzed using the statistical package for the social sciences (SPSS) version 16.0 or higher (SPSS, Chicago, Ill., USA). Normality of continuous data was tested with the Shapiro–Wilk test, and homogeneity of variances was tested using the Levene’s test. Descriptive analysis was performed in order to describe baseline characteristics (intrinsic, injury, and intervention-related variables) and outcome measures. Continuous data are reported as medians and percentiles (nonparametric data) or as means and standard deviation (parametric data), and categorical data as numbers with percentages. A Mann–Whitney *U* test (numeric variables) or chi-squared analysis was performed in order to assess whether there were differences in characteristics and outcome if surgery was performed on the dominant side versus the nondominant side. We also assessed whether outcome differed between the two hospitals. A multivariable linear regression analysis was performed in order to model the relation between different covariates and the Constant score. Intrinsic, injury, and intervention-related variables were added as covariate. Similar models were made for the other numeric outcome measures.

## Results

### Patient and intervention characteristics

Sixty patients underwent a modified Putti-Platt procedure between 2000 and 2010, and nine patients could not be retrieved (*N* = 7) or did not consent to participate (*N* = 2). The remaining 51 patients, of which 37 (73 %) were male, could be enrolled after a median follow-up time of 4.7 (P_25_–P_75_ 1.7–6.8) years (Table 1). All of these patients completed every questionnaire. Most patients sustained their initial dislocation during sporting activities (*N* = 30, 59 %). Intervention characteristics are also outlined in Table 2. Of all patients’ standard shoulder X-rays were made prior to surgery. In a subset of patients, additional MRI scans (*N* = 33, 65 %) or CT scans (*N* = 8, 16 %) were made. Thirty-one (61 %) patients had radiological signs of a Hill-Sach’s lesion, none of which required surgical repair. Forty-five patients (88 %) had a Bankart lesion (Table 2), and three patients (6 %) had SLAP lesions. All labral lesions were repaired upon recognition with a median of two (P_25_–P_75_ 2–3) suture anchors. Unfortunately, intra-operative range of motion was only rarely recorded. No intra-operative complications were encountered. Eight patients (16 %) developed recurrent instability; five of these patients (10 %) reported a recurrent dislocation after 6, 5, 3, and 1 year, respectively. For the fifth patient, the dislocation date was not recorded. Multivariable binary logistic regression analysis showed a positive relation between the occurrence of a recurrent dislocation after surgery (dependent variable) and the duration of the post-operative period in years. The adjusted Exp(b) value, after correction for age, duration of symptoms prior to surgery, surgery of the dominant side, and gender, was 1.806 (95 % CI 1.077–3.029; *p* = 0.025;) per year. Two patients with recurrent dislocations required a secondary intervention; one patient underwent a second-modified Putti-Platt procedure, and the other patient was treated with a Bristow-Latarjet procedure. Both patients currently have a stable shoulder. The three patients who did not experience actual re-dislocations reported subluxation or complained of subjective instability. Patient and intervention characteristics did not differ between the two hospitals.

**Table 1:** Baseline characteristics of the study population

Total number of patients	51
Male^a^	37 (72.5)
Age at first dislocation (year)^b^	21 (13–24)
Age at surgery (year)^b^	25 (21–39)
Time between first dislocation and surgery (months)^b^	24 (12–96)
Length of follow-up (months)^b^	56 (20–81)
Right side affected*	28 (54.9)
Dominant side affected*	25 (55.6)
*Total number of dislocations* ^a^	
<5	23 (45.1)
5–10	14 (27.5)
10–15	5 (9.8)
>15	9 (17.6)
*Trauma mechanism* ^*a*^	
Low-energy trauma; fall from standing height	3 (5.8)
High-energy trauma	8 (15.6)
Sports	30 (58.8)
Assault	3 (5.8)
Pulling or lifting	4 (7.8)
Other	3 (5.8)
Smoking at time of surgery^a^	21 (41.2)
Alcohol consumption at time of surgery^a^	37 (72.5)

* In six patients the dominance was unknown, therefore this percentage was calculated for 45 patients instead of 51

Data are shown as ^a^ numbers with percentages or as ^b^ median with P_25_–P_75_ between brackets

**Table 2:** Pathologic lesions and intervention characteristics

Bankart lesion^a^	45 (88.2)
Labral tear	33 (73.3)
Bony	12 (26.7)
SLAP lesion^a^	3 (5.9)
Capsule tear	3 (5.9)
Hill-Sachs lesion^a^	31 (60.8)
Suture anchors^a^	48 (94.1)
Number^b^	2 (2–3)

Data are shown as ^a^ numbers with percentages or as ^b^ median with P_25_–P_75_ between brackets

### Range of motion and strength

A median loss of seven (P_25_–P_75_ 0–15) degrees of abduction and six (P_25_–P_75_ 0–10) degrees of elevation was measured when comparing the affected side with the contralateral side (Table 3). A median loss of 10 (P_25_–P_75_ 0–20) degrees of external rotation and eight (P_25_–P_75_ 0–15) degrees of external rotation in 90° of abduction was observed. The motion restriction was consistently lower when the dominant side was affected than when the nondominant side was affected (*p* > 0.05). The median strength of abduction as measured at an arm’s length in 90° of abduction was 7.5 (P_25_–P_75_ 6.0–10.0) kg for the operated arm versus 9.5 (P_25_–P_75_ 7.0–11.0) kg for the contralateral arm. Range of motion and strength was not significantly different when comparing the two hospitals.

**Table 3:** Functional outcome, quality of life, and patient satisfaction with the result of the modified Putti-Platt procedure

	Overall (*N* = 51)	Dominant side affected (*N* = 25)*	Nondominant side affected (*N* = 20)*
*Loss of ROM* *(degrees)* ^a^			
Abduction	7 (0–15)	5 (0–8)	10 (0–22)
Elevation	6 (0–10)	6 (0–10)	9 (0–10)
External rotation	10 (0–20)	10 (0–20)	13 (0–29)
External rotation in abduction	8 (0–15)	7 (0–10)	12 (0–24)
*Constant score*			
Affected side	84 (75–91)	83 (75–93)	89 (83–93)
Contralateral side	92 (84–95)	92 (86–97)	95 (87–98)
Percentage of unaffected arm	94 (88–99)	91 (91–97)	95 (91–97)
*DASH score*			
Total	5.0 (0.8–10.8)	5.0 (0.8–10.4)	1.6 (0.2–11.0)
Work	0.0 (0.0–15.6)	0.0 (0.0–9.4)	0.0 (0.0–0.0)
Sports/Music	0.0 (0.0–25.0)	0.0 (0.0–7.8)	6.3 (0.0–75.0)
*Rowe score*			
Total	92 (75–95)	95 (75–95)	92 (75–95)
Stability	50 (50–50)	50 (50–50)	50 (50–50)
ROM	15 (15–15)	15 (15–15)	15 (5–15)
Function	30 (25–30)	30 (25–30)	30 (25–30)
*WOSI*			
Total score	8.9 (6.8–9.4)	8.8 (6.8–9.4)	9.1 (7.9–9.5)
Physical	8.8 (7.5–9.5)	8.7 (7.5–9.5)	9.1 (7.8–9.5)
Sports/recreation/work	8.4 (6.3–9.5)	8.5 (6.4–9.5)	8.4 (6.5–9.5)
Lifestyle	8.5 (7.5–9.5)	8.4 (7.3–9.6)	9.1 (7.8–9.6)
Emotion	8.8 (6.8–9.4)	8.0 (7.2–9.4)	9.1 (7.3–9.6)
VAS for patient satisfaction	7.9 (6.8–9.5)	8.0 (6.8–9.6)	7.9 (7.3–9.8)

Data are shown for all patients, for patients whose dominant side was affected and for patients whose nondominant side was affected

Data are shown as median with P_25_–P_75_ between brackets. Differences between both groups were assessed using the Mann–Whitney *U* test. In all tests, the *p* value was >0.050

*DASH* disabilities of the arm, shoulder and hand, *ROM* range of motion *VAS* visual analog score, *WOSI* Western Ontario shoulder index

* In six patients the dominance was unknown, therefore this percentage was calculated for 45 patients instead of 51

^a^Data were expressed as differences in ROM of the operated minus the nonoperated side

### Functional outcome and quality of life

Overall, the median DASH score was 5.0 (P_25_–P_75_ 0.8–10.8) indicating very little disability. Patients whose dominant side was affected scored 3.4 points more than patients whose nondominant side was affected (Table 3). Both subgroups reported a median score of 0.0 in the high performance section for work. Patients whose dominant side was affected also reported a median of 0.0 points in the high performance section for sports/music, whereas patients whose nondominant side was affected scored 6.3 (P_25_–P_75_ 0.0–75) points.

The median Constant score for the whole group was 84 (P_25_–P_75_ 75–91) points. Furthermore, the relative Constant score was calculated as the score of the affected arm as a percentage of the patient’s healthy arm; this was 94 % (P_25_–P_75_ 88–99).

A median ROWE score of 92 (P_25_–P_75_ 75–95) was measured for the whole group, which is considered as an excellent result. Forty patients (78 %) scored more than 74 points, which is the threshold for a good or excellent result.

The median WOSI score was 8.9 (P_25_–P_75_ 6.8–9.4) with none of the sub-domains (physical, sports/recreation/work, lifestyle, and emotions) scoring a loss of injury-related quality of live greater than 10 % (median). Scores were similar in patients whose dominant or nondominant shoulder had been treated. The overall SF-36 score was 107.7 (P_25_–P_75_ 93.0–113.1). Both the physical and mental components of the SF-36 were within the population norm of 50 ± 10 (SD) points and were independent of the affected side. Patients reported a high satisfaction with the outcome on the VAS score; 76 % of all patients scored seven or more points out of 10. Functional scores and quality of life were not significantly different when comparing the two hospitals.

## Discussion

Our results show that the modified Putti-Platt procedure, as performed in our case series, is an effective treatment for recurrent anterior shoulder instability, leading to acceptable recurrence rates and satisfactory functional outcome, quality of life, and patient satisfaction.

In the literature, an anterior labral detachment (i.e., Bankart lesions) is present in approximately from 65 to 90 % of the shoulders that are surgically treated for anterior instability [4, 10, 20, 23]. In 88 % of the patients in this study, a Bankart lesion was present and subsequently treated. Bankart lesions contribute to recurrent shoulder instability [24–26]. Nevertheless, most modifications of the original Putti-Platt procedure do not address any contributing anatomic pathology (i.e., labrum and glenoid pathology) apart from capsular laxity and subscapularis muscle redundancy. On the other hand, despite their important roles in shoulder instability, capsular laxity and subscapularis muscle redundancy are neglected subjects in most reports on Bankart repairs [20, 27]. For these reasons, both aspects of glenohumeral instability were addressed in the current study. Several authors have described Bankart repairs in combination with a capsular shift procedure [9, 20, 28]. However, shortening of the subscapularis muscle is often not performed. Only one retrospective report of 30 patients was found in which all three aspects of shoulder instability were addressed [29].

Although most surgically stabilized shoulders remain stable over time, recurrent dislocations are important complications to take into account. Recurrent dislocations occurred in 10 % of our patients, which is in line with the 10 % found by Pelet et al. [29] who used a similar technique. Hayes et al. [1] reported a mean re-dislocation rate of 11 % after a mean follow-up of 4.3 years in seven studies following open Bankart repair. Re-dislocation rates of up to 36 %, most of which were higher than 20 %, have been reported in several studies after a Putti-Platt procedure [9, 30–33]. Recurrences tend to occur even after a longer post-operative time [6, 34]. However, most recurrent dislocations occur within the first 5 years following surgery [29].

Arthroscopic treatment has evolved greatly over the past decades, gaining in interest over open procedures. Potential benefits of minimally invasive procedures include less surgical dissection and post-operative pain and an improved range of motion. However, in a meta-analysis on open versus arthroscopic stabilization by Lenters et al. [35], 97 of the 527 (18 %) arthroscopically treated patients experienced recurrent stability. A meta-analysis from 2004 studying the same subject also demonstrated a higher recurrence rate in patients treated arthroscopically (3 vs. 13 %) [36]. Another meta-analysis from 2010 on this topic found a recurrence rate of only 2.9 % in the arthroscopic group when only including trials from later than 2002 [37]. This suggests that arthroscopic techniques have evidently improved over time. Unfortunately, functional outcome and range of motion could not be adequately addressed.

Particularly, loss of range of motion has been labeled as the greatest disadvantage of the original procedure. Even after the modification as described by Symeonides et al., restrictions in external rotation of up to 29° have been reported [10, 38, 39]. Pelet et al. [29] who used a different modification even found a mean loss of 33° of external rotation and a mean loss of 24° loss of external rotation in 90° of abduction (*N* = 39] as opposed to 10° and 8°, respectively, in the current study. The limited restriction of external rotation as encountered in the current study could be explained by the fact that extra care was taken not to over-shorten the subscapularis muscle. Another explanation could lie in the duration of follow-up since surgery. The restriction in ROM has proven to diminish during the course of the post-operative period [38, 40].

The median DASH score in our population was 5.0 points, which is comparable to the 4.3 points that Hovelius et al. [41] found in 17 patients, 25 years after a (simplified) Putti-Platt procedure. The slightly inferior result for the Constant score was largely attributed to a median difference in strength of 2.0 kg between the affected and healthy shoulder. To the best of our knowledge, no previous studies have reported on the health-related quality of life after stabilizing procedures of the glenohumeral joint.

All procedures in these series were performed by three surgeons. This could be considered as a drawback that might have introduced a bias, but also as strength because all procedures were performed in a consistent way. The fact that satisfactory results were obtained in most patients, irrespective of the hospital where the surgery was performed, emphasizes the generalizability of this modification of the Putti-Platt procedure. A limitation of this study is that we performed multiple statistical comparisons on a relatively small population, which has a risk of accepting a spurious relation. However, applying the Bonferroni correction would be too stringent and might falsely reject true effects.

Ahmad et al. [24] stated that “the ideal surgical goals in treating shoulder instability are to anatomically correct all of the contributing pathology encountered, preserve range of motion, and preserve or restore normal joint mechanics”. Our data show that the modified Putti-Platt procedure as performed in our series closely meets these criteria; motion restriction is marginal, outcome scores are satisfactory, and the patients are highly satisfied with the end result. The modified Putti-Platt procedure can therefore be considered as an effective treatment option for recurrent anterior traumatic shoulder instability.
